# Long noncoding RNA CASC9/miR‐519d/STAT3 positive feedback loop facilitate the glioma tumourigenesis

**DOI:** 10.1111/jcmm.13932

**Published:** 2018-09-30

**Authors:** Hongjiang Liu, Chen Li, Jiankai Yang, Yuchen Sun, Shunyao Zhang, Jipeng Yang, Liang Yang, Yuanyu Wang, Baohua Jiao

**Affiliations:** ^1^ Department of Neurosurgery The Second Hospital of Hebei Medical University Shijiazhuang Hebei China; ^2^ Department of Pathology at Basic Medical College of Guizhou Medical University Guiyang Guizhou China; ^3^ Department of Neurosurgery Affiliated Hospital of Guizhou Medical University Guiyang Guizhou China

**Keywords:** CASC9, feedback loop, glioma, miR‐519d, STAT3

## Abstract

Emerging evidence have illustrated the vital roles of long noncoding RNAs (lncRNAs) in glioma. Nevertheless, the majority of their roles and mechanisms in gliomagenesis are still largely unclear. In this study, we investigate the roles of lncRNA CASC9 on glioma tumourigenesis and authenticate its potential mechanisms. Results manifested that CASC9 was highly expressed in glioma specimens and cells, moreover, the ectopic overexpression was correlated with glioma patients’ clinic. Functional studies found that siRNA‐mediated CASC9 silencing inhibited the proliferative ability, invasion in vitro, and impaired the tumour growth in vivo. Mechanical studies revealed that miR‐519d both targeted the 3′‐UTR of CASC9 and STAT3 mRNA, which was identified by luciferase reporter assay and RNA immunoprecipitation (RIP). Moreover, chromatin immunoprecipitation (ChIP) and luciferase reporter assay revealed that STAT3, an oncogenic transcription factor, could bind with the promoter of CASC9 and activate its transcriptional level. In conclusion, our results concluded that CASC9 promotes STAT3 expression via sponging miR‐519d, in return, STAT3 activate CASC9 transcription, forming a positive feedback loop of CASC9/miR‐519d/STAT3. The novel finding provides a potential therapeutic target for glioma.

## 1 INTRODUCTION

Glioma is one of the most common human malignant tumour, moreover, it acts as the aggressive tumour and primary brain tumour in the central nervous system.^1^, ^2^ Glioma, accounting for 70% of brain tumours, causes thousands of cancer‐related death worldwide. Despite the combination therapy, eg, surgical excision, chemotherapy, and radiotherapy, have significantly improved the long‐term curative effect and prognosis, the survival rate and curative ratio is still pessimistic.^3^ Glioma is characterized by high invasive and diffusion, yet the potential mechanisms remain far from understanding.^4^ So, it is urgently needed to discover the molecular mechanisms of glioma tumourigenesis.

Long noncoding RNAs (lncRNAs) are a group of RNA with longer than 200 nucleotides, however, without protein‐coding potential. In decades, noncoding RNAs (ncRNAs) are considered as the transcriptional byproducts without biological roles on pathophysiological processes.^5^, ^6^, ^7^ However, with the help of next‐generation sequencing (NGS) and bioinformatics tools, the crucial roles of lncRNAs have been wildly recognized.^8^, ^9^ The dysregulation of lncRNAs regulates the transcriptional regulation or post‐transcriptional regulation. For instance, lncRNA LINC00958 is significantly up‐regulated in glioma tissues and cell lines compared with that of adjacent normal brain tissues, and the overexpression acts as an oncogenic gene in the gliomagenesis through miR‐203‐CDK2 regulation.^10^ LncRNA HOXA11‐AS is up‐regulated in glioma tissue and cell lines and the high HOXA11‐AS expression indicates shorter survival and poorer prognosis.^11^

It has been clearly reported that lncRNA CASC9 (Cancer Susceptibility Candidate 9) have been identified to contribute to the initiation and progression of several human cancers, including oesophageal cancer, lung adenocarcinoma and so on, involving in proliferation, invasion, metastasis.^12^, ^13^ In present study, our team aim to uncover the role of CASC9 in the glioma tumourigenesis, presenting the ectopic overexpression of CASC9 in the progression by which accelerates the gliomagenesis by targeting miR‐519d/STAT3 positive feedback loop.

## 2 MATERIALS AND METHODS

### 2.1 Clinical tissue samples

Clinical tissue samples (28 samples) were enrolled from glioma patients who undergoing surgical excision at The Second Hospital of Hebei Medical University. Before the surgery, all the patients were aware of the study and provided signed informed consent. Glioma tissues and paired adjacent normal tissues were collected immediately after resection and stored in liquid nitrogen before further use.

### 2.2 Cell culture and small interfering RNA (siRNA) synthesis

The human glioma cell lines (SHG44, U87, U251, A172) and normal human astrocytes (NHA) were purchased from the American Type Culture Collection (ATCC, USA) and cultured RPMI‐1640 medium (Gibco, Carlsbad, CA, USA) supplemented with 10% foetal bovine serum (FBS, Gibco, USA) and 100 U/mL penicillin/streptomycin (Life Technologies, USA) in a humidified incubator at 37°C with 5% CO_2_. Small interfering RNA (siRNA) were designed and synthesized by Shanghai GenePharma Company (Shanghai, China). siRNA targeting CASC9 (si‐CASC9) and negative control (50 n mol L^−1^) were separately transfected into cells using Lipofectamine 2000 reagent (Invitrogen, USA). The sequences of si‐CASC9 were presented as following: si‐CASC9‐1, 5′‐GUGUCUGCAACAACUUUAAUU‐3′, si‐CASC9‐2:5′‐UUACAGAGUUAAUUGGCACUU‐3′, si‐CASC9‐3, 5′‐GUAAGAACAACUUCUGCUUUU‐3′.

### 2.3 RNA extraction and quantitative real‐time PCR

Total RNA was isolated from glioma tissue and cells utilizing TRIzol reagent (Invitrogen) according to the manufacturer's instructions. Complementary DNA (cDNA) was reversely transcribed from RNA (1 μg). The reverse transcription PCR reactions were performed using a standard SYBR Green PCR kit (TaKaRa, Dalian, China) on Applied Biosystems 7300 Real‐Time PCR system (Applied Biosystems). GAPDH serviced as an internal control. The relative expression levels (fold change) were calculated by the 2^−▵▵Ct^ method and every data were performed in triplicate. These primers included: CASC9, forward, 5′‐AGATGAAGCCGGTACCTCAGAT and 5′‐TCACTTTAAAGAGGGAGAGGAG‐3′; GAPDH, forward, 5′‐AGCCACATCGCTCAGACAC‐3′ and reverse, 5′‐GCCCAATACGACCAAATCC‐3′.

### 2.4 Cellular proliferation assays

The proliferation assay was conducted using CCK‐8 assay and colony formation assay. Cell viability was tested by Cell Counting Kit‐8 (Dojindo) to the manufacturer's instructions. For colony formation, cells (1 × 10^3^) were resuspended in medium and plated onto a bottom layer. After 14 days, the clones that larger than 0.5 mm were counted.

### 2.5 Transwell invasion assay

The invasive ability of glioma cells was evaluated using transwell chambers (8 μm, Corning Life Sciences, Corning, NY, USA) polycarbonate membrane. Briefly, cells (3 x 10^4^ cells) were seeded above the membrane of chamber added with serum‐free DMEM. The lower chamber was added with 600 μl DMEM supplemented with 10% FBS. After culture at 37°C for 48 hours, cells on the upper chamber were removed. The invaded cells were fixed with ethanol. After 0.2% crystal violet stained, the cells were counted under a microscope.

### 2.6 Western blot analysis

Glioma cells lysates were prepared with RIPA Lysis buffer (Beyotime, Shanghai, China) containing protease inhibitor cocktail (Roche, Mannheim, Germany). Protein concentration was measured by Pierce BCA Protein Assay Kit (Beyotime) and then subjected to sodium dodecyl sulphate polyacrylamide gel electrophoresis (SDS‐PAGE). Then, proteins were transferred into PVDF membrane and blocked using 5% fat‐free milk. The members were incubated with primary anti‐STAT3 (dilution 1:1000, Abcam) and anti‐GAPDH (dilution 1:1000, Abcam) antibody. Finally, membrane was incubated with secondary antibody (HRP labelled goat anti‐rabbit IgG, 1:2000, CST) and measured using ECL detection system (Thermo Fisher, Rockford, IL, USA) and Quantity One software (Bio‐Rad, Hercules, CA, USA).

### 2.7 Subcellular fractionation assay

The RNA was extracted from nuclear or cytoplasm fraction was measured using RT‐qPCR analysis. U6, GAPDH acted as the nuclear or cytoplasm fractions using Nuclear and Cytoplasmic Extraction Reagents (Thermo Scientific, USA).

### 2.8 Dual‐luciferase reporter assay

The sequences containing the wild‐type or mutant sites targeting miR‐99 of CASC9 and STAT3 3′‐Untranslated Region (UTR) were cloned into the pGL3‐basic vector (Promega, USA) to construct the pGL3‐CASC9 wild/mutant, and pGL3‐STAT3 wild/mutant. Besides, the sequences containing CASC9 promoter region was also cloned into the pGL3‐basic vector (Promega). All reporter vectors were cotransfected with miRNAs or negative controls into 293T cells. After 48 hours, luciferase activity was measured using the Dual Luciferase Reporter Assay System (Promega).

### 2.9 RNA immunoprecipitation

RIP assay was done following Magna‐RIP RNA Binding Immunoprecipitation Kit (Millipore, Bedford, MA, USA) as previous described. Briefly, cells lysis was eluted and incubated with RIP buffer. Anti‐Ago2 antibody (Millipore) or negative control normal Mouse IgG (Millipore) was conjugated with magnetic beads. Subsequently, the enrichment in immunoprecipitation was measured using PCR.

### 2.10 Chromatin immunoprecipitation

ChIP assay was conducted according to EZ ChIP Chromatin Immunoprecipitation Kit (Millipore, Bedford, MA, USA) as prior described. Briefly, anti‐STAT3 antibody (Millipore) was used to immunoprecipitate the chromatin, and immunoglobulin G (IgG) was used as negative control. Lastly, the enrichment of isolated RNA was tested using RT‐PCR,

### 2.11 In vivo xenograft assay

BALB/c nude mice (4‐5 weeks) were purchased and maintained under specific pathogen‐free conditions. About 1 × 10^7^ cells/ml (0.1 ml) glioma cells (U87) were subcutaneously injected into flank of mice. Tumour volume was calculated according to the following formula: 0.5 × (longest diameter × shortest diameter^2^). The research was approved by Animal Care and Use Committee of the Second Hospital of Hebei Medical University, and in strict accordance with the Care and Use of Laboratory Animals of the National Institutes of Health.

### 2.12 Statistical analysis

All results are presented as the mean ± SD by three independent assays. The significance between two groups was calculated using student's *t* test and one way ANOVA. Survival rate was calculated using Kaplan‐Meier analysis and log‐rank test. *P*‐values <0.05 was considered as statistically significant.

## 3 RESULTS

### 3.1 LncRNA CASC9 is highly expressed in glioma tissue

**Figure 1:**
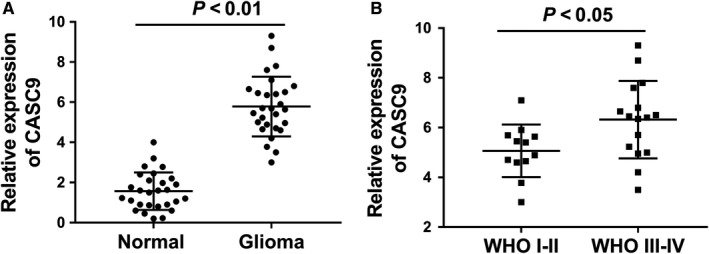
LncRNA CASC9 is highly expressed in glioma tissue. A, LncRNA CASC9 expression was identified in glioma tissue and adjacent normal tissue specimen using RT‐PCR. B, LncRNA CASC9 expression in advanced pathological type (WHO III‐IV) and primary pathological type (WHO I‐II)

As a first attempt to explore whether lncRNA CASC9 participated the pathogenesis of glioma, we measured the expression level of lncRNA CASC9 to identify whether it was aberrantly expressed in glioma tissue specimen. Results revealed that CASC9 expression was markedly overexpressed in the collected glioma tissue specimens compared with adjacent normal tissue (Figure 1A). Among these glioma tissue specimens, CASC9 expression was markedly up‐regulated in advanced pathological type (WHO III‐IV) than that of primary pathological type (WHO I‐II) (Figure 1B). In conclusion, our results indicate that lncRNA CASC9 is highly expressed in glioma tissue.

### 3.2 LncRNA CASC9 silencing impaired the proliferation, invasion in vitro, and inhibited the tumour growth in vivo

**Figure 2:**
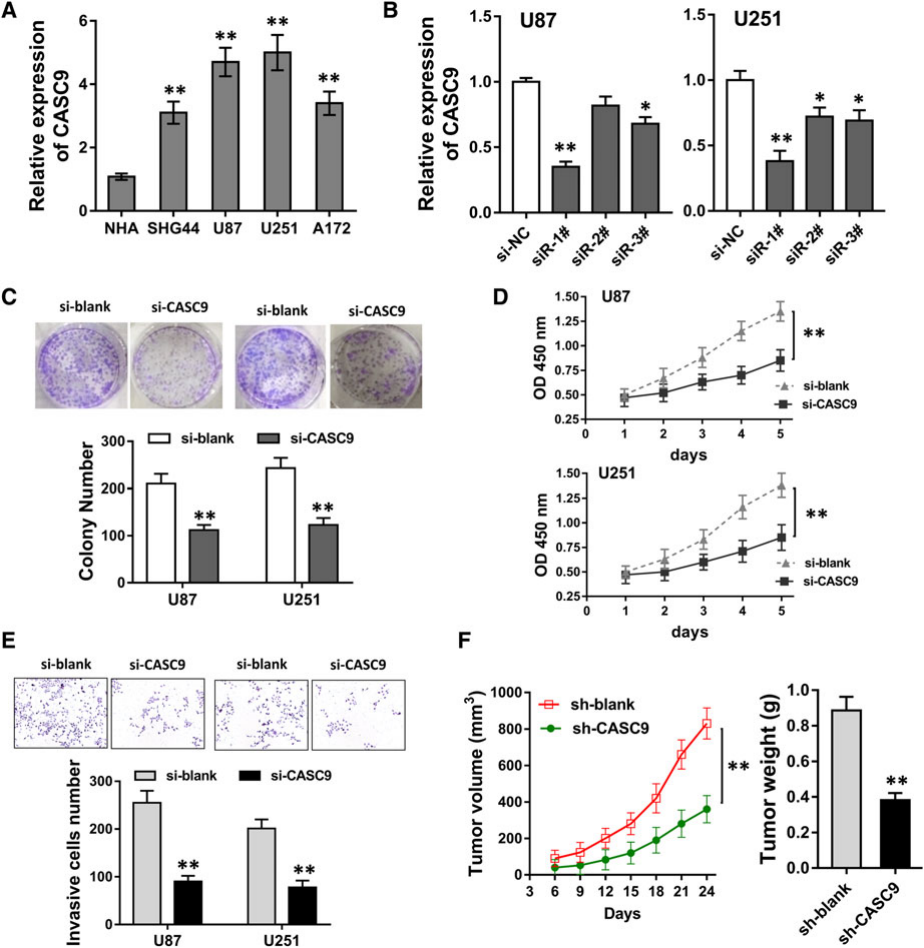
CASC9 silencing impaired the proliferation, invasion in vitro, and inhibited the tumour growth in vivo. A, RT‐PCR revealed the lncRNA CASC9 expression in glioma cell lines. B, siRNAs targeted CASC9 were transfected into U87 and U251 cells to silence its expression. C, Clone formation assay illustrated the clone number in U87 and U251 cells of CASC9 silencing or controls. D, CCK‐8 assay revealed the absorbance or proliferative abilities in U87 and U251 cells of CASC9 silencing or controls. E, Transwell invasive assay illustrated the invaded cells number in U87 and U251 cells of CASC9 silencing or controls. F, In vivo xenograft mice assay illustrated the tumour volume and weight of CASC9 stable silencing by lentivirus. *P < 0.05, **P < 0.01 compared to control group

Clinically, we found that CASC9 was highly expressed in glioma tissue. Thus, in the following cellular experiments, we performed a series of loss‐of‐functional experiments to identify the biological functions of lncRNA CASC9. RT‐PCR revealed that lncRNA CASC9 expression was significantly up‐regulated in glioma cell lines (Figure 2A). siRNAs targeted CASC9 were transfected into U87 and U251 cells to silence its expression (Figure 2B). Clone formation assay illustrated that CASC9 silencing decreased the clone number in U87 and U251 cells compared with the controls (Figure 2C). CCK‐8 assay revealed that CASC9 silencing inhibited the absorbance or proliferative abilities in U87 and U251 cells compared with the controls (Figure 2D). Transwell invasive assay illustrated that CASC9 silencing inhibited the invaded cells number compared with the controls (Figure 2E). In vivo xenograft mice assay illustrated that CASC9 stable silencing by lentivirus could markedly decreased the tumour volume and weight (Figure 2F). Therefore, results revealed that CASC9 silencing impaired the proliferation, invasion in vitro, and inhibited the tumour growth in vivo.

### 3.3 miR‐519d directly targeted the 3′‐UTR of CASC9

**Figure 3:**
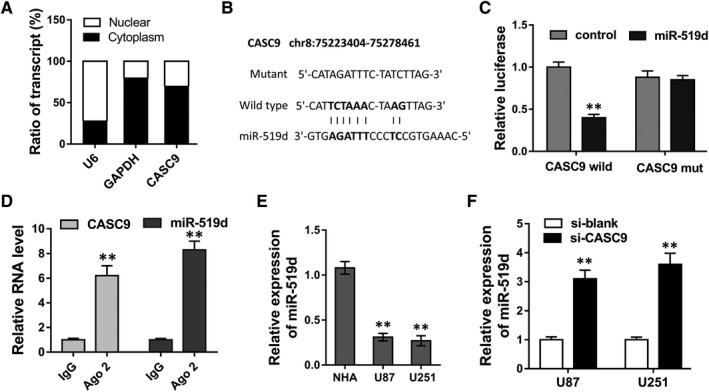
miR‐519d directly targeted the 3′‐UTR of CASC9. A, Subcellular fractionation assay revealed the subcellular location of CASC9 U87 cells. B, Complementary binding sites of miR‐519d with the 3′‐UTR of CASC9 predicted by bioinformatics online tools. C, Luciferase activity assay was conducted to confirm the binding of miR‐519d with the 3′‐UTR of CASC9. D, Ago2 RIP assay revealed the enrichment of miR‐519d and CASC9 in RNA‐induced silencing complex (RISC). E, RT‐PCR revealed the miR‐519d expression level in U87 and U251 cells. F, RT‐PCR revealed the miR‐519d expression level in U87 and U251 cells transfected with si‐CASC9. *P < 0.05, **P < 0.01 compared to control group

To identify the underlying mechanism that CASC9 accelerated the tumourigenesis of glioma, we sequentially performed mechanical experiments. Subcellular fractionation assay revealed that the subcellular location of CASC9 was mainly located in cytoplasm (Figure 3A). Bioinformatics online tools (LNCipedia, https://lncipedia.org/) manifested that there were several complementary binding sites of miR‐519d with the 3′‐UTR of CASC9 (Figure 3B). To confirm the binding, luciferase activity assay was conducted and revealed that miR‐519d could directly target with the 3′‐UTR of CASC9 (Figure 3C). Following RIP assay revealed that miR‐519d and CASC9 were both enriched in the immunoprecipitation, suggesting the incorporation within RNA‐induced silencing complex (RISC) (Figure 3D). RT‐PCR revealed that miR‐519d expression level was decreased in U87 and U251 cells (Figure 3E), besides, it was increased in U87 and U251 cells transfected with si‐CASC9 (Figure 3F). Overall, data revealed that miR‐519d directly targeted the 3′‐UTR of CASC9.

### 3.4 STAT3 acted as the target of miR‐519d/CASC9

**Figure 4:**
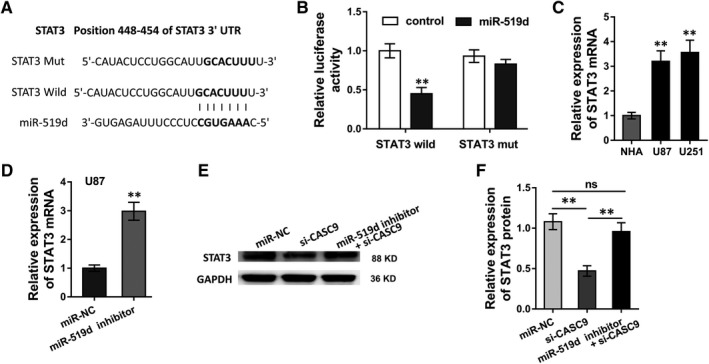
STAT3 acted as the target of miR‐519d/CASC9. A, Complementary binding sites of miR‐519d with the 3′‐UTR of STAT3 mRNA predicted by bioinformatics online tool (TargetScan, http://www.targetscan.org). B, Luciferase activity assay was conducted to confirm the binding of miR‐519d with the 3′‐UTR of STAT3 mRNA. C, RT‐PCR analysis illustrated the STAT3 mRNA expression level in U87 and U251 cells. D, RT‐PCR analysis illustrated the STAT3 mRNA in U87 cells transfected with miR‐519d inhibitor. (E, F) Western blot analysis illustrated the STAT3 protein level in U87 cells co‐transfected with si‐CASC9 and/or miR‐519d inhibitor. *P < 0.05, **P < 0.01 compared to control group. ns presented no significant difference

In order to test the role of miR‐519d and CASC9 on glioma, we further discovered the downstream target of miR‐519d. Bioinformatics tools (TargetScan, http://www.targetscan.org) revealed that STAT3 might function as the downstream target of miR‐519d utilizing the complementary binding sites (Figure 4A). To confirm the binding, luciferase activity assay was conducted and revealed that miR‐519d could directly target with the 3′‐UTR of STAT3 mRNA (Figure 4B). RT‐PCR analysis illustrated that STAT3 mRNA expression level was markedly increased in U87 and U251 cells (Figure 4C). In U87 cells, STAT3 mRNA expression level was markedly increased when transfected with miR‐519d inhibitor (Figure 4D). Western blot analysis revealed that si‐CASC9 transfection could significantly decreased the STAT3 protein level, while the cotransfection of si‐CASC9 and miR‐519d inhibitor recovered the expression of STAT3 protein (Figure 4E,F). Overall, these evidence could powerfully supported that STAT3 acted as the target of miR‐519d/CASC9, suggesting the pathway of CASC9‐miR‐519d‐STAT3.

### 3.5 STAT3 accelerated CASC9 expression through binding with its promoter

**Figure 5:**
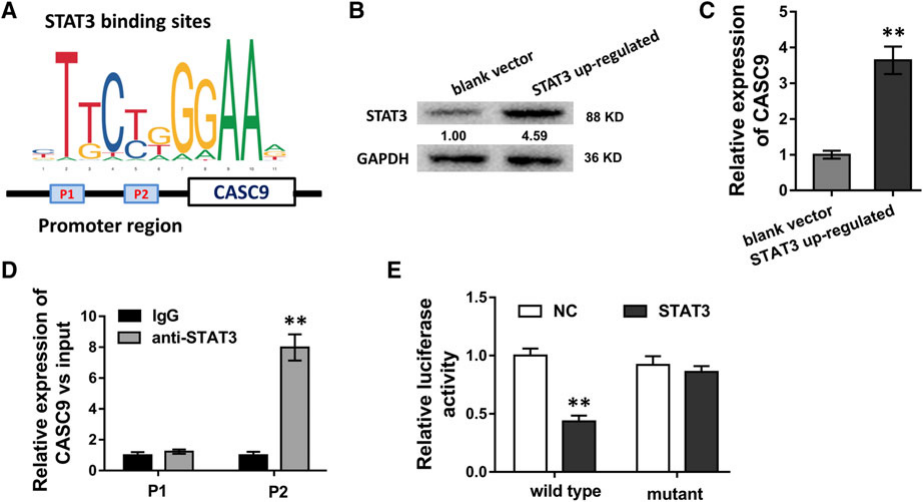
STAT3 accelerated CASC9 expression through binding with its promoter. A, The schematic diagram showed the binding within STAT3 and CASC9 promoter region, including two predicted sites (P1, P2). B, STAT3 protein expression level in U87 cell transfected with STAT3 recombinant plasmid. C, RT‐PCR revealed the CASC9 expression in U87 cell transfected with STAT3 recombinant plasmid. D, ChIP analysis showed the binding affinity in two candidate sites (P1, P2). E, Luciferase reporter assay revealed the activity within STAT3 and CASC9 wild‐type or mutant. *P < 0.05, **P < 0.01 compared to control group

In subsequent experiments, we continued to investigate whether transcription factor STAT3 was closely correlated with lncRNA CASC9. Based on Jaspar (http://jaspar.genereg.net), we predicted that STAT3 had potential binding sites with CASC9 promoter, including two binding sites with high possibility at the upstream of CASC9 (about −2000 to +1 bp) (Figure 5A). In U87 cell, the up‐regulated STAT3 recombinant plasmid could markedly increase STAT3 protein expression level (Figure 5B). RT‐PCR revealed that STAT3 up‐regulation could increase CASC9 expression (Figure 5C). ChIP analysis showed that the binding affinity in second site (P2) is significantly higher, indicating the direct binding within STAT3 and CASC9 promoter (Figure 5D). Luciferase reporter assay revealed that CASC9 wild‐type had high activity with STAT3 (Figure 5E). Therefore, results concluded that STAT3 accelerated CASC9 expression through binding with its promoter.

## 4 DISCUSSION

Growing evidence have revealed that approach 3000 human lncRNAs are exist in the genome, however, only less than 1% have been characterized.^14^ Numerous literature have indicated that lncRNAs were vital elements in human tumourigenesis. LncRNA CASC9 (Cancer Susceptibility Candidate 9) have been identified to contribute to the initiation and progression of several human cancers, including oesophageal cancer, lung adenocarcinoma, and so on.^12^, ^13^

The increasing vital roles of lncRNAs have been involved in more and more extensive fields, including human tumours, cardiovascular system disorder, or metabolic diseases.^15^, ^16^, ^17^, ^18^, ^19^ In this study, our data concluded that CASC9 was dramatically high‐expressed in glioma tissue and cells, illustrating the pessimistic role of CASC9 in glioma patients. Clinically, the aberrant overexpression of CASC9 was correlated with the clinical of glioma patients, implying the tumour promoting biomarker of CASC9. More than CASC9, numerous of lncRNAs have been identified to be aberrantly overexpressed in human tumourous tissue, acting as the oncogenic molecular, such as PVT1,^20^ UCA1,^21^ CCAT2^22^ et al. Similar to this finding, our results also discover this worthy conclusion.

The clinical or phenotype finding of CASC9, as a potential oncogenic RNA, has been determined in previous assays. Subsequently, the cellular biologic roles of lncRNA CASC9 are identified in loss‐of‐functional experiments. Results illustrated that CASC9 silencing impaired the proliferation, invasion in vitro, and inhibited the tumour growth in vivo. Therefore, the cellular functions of CASC9 are clearly considered as oncogene in glioma tumourigenesis. We confirmed that CASC9 is not only closely correlated with the clinical phenotype of glioma patients, but also involved in the molecular level of cells’ invasion and metastasis. More than CASC9, a large number of lncRNAs have been reported. For instance, lncRNA HOTTIP is up‐regulated in glioma cells treated by hypoxia, and knockdown of HIF‐1α and HOTTIP blocked hypoxia‐induced EMT, and suppressed invasion and migration of glioma cells by regulating the miR‐101/ZEB1 axis.^23^ HOXA11‐AS is significantly up‐regulated in glioma tissues and cell lines and its knockdown inhibits glioma cell proliferation, migration and invasion in vitro, and tumour growth in vivo, thereby via ceRNA for miR‐214‐3p and its direct target EZH2.^24^

Excepting for the molecular role of CASC9, the mechanical interaction is still attractive. Firstly, the subcellular location of CASC9 is in the cytoplasm, indicating the post‐transcriptional control of CASC9 in the glioma cells. Interestingly, we found that CASC9 harbours complementary binding sites with miR‐519d at its 3′‐UTR. The mechanism of CASC9 and miR‐519d is regarded as competing endogenous RNA (ceRNA). CASC9 acts as a sponge of miR‐519d in the cellular mechanism. For example, lncRNA MEG3 inhibited miR‐93 level and the expression of PI3K/AKT pathway related proteins, forming the MEG3‐miR‐93‐PI3K‐AKT pathway in glioma cells.^25^

Interestingly, we found that STAT3 protein acted as the target of miR‐519d, constituting the CASC9‐miR‐519d‐STAT3 axis. STAT3 acts as a transcription factor.^26^, ^27^, ^28^, ^29^ With the assistance of bioinformatic JASPAR tool, STAT3 could also bind with the promoter region of lncRNA CASC9, in turn, promoting the transcription activity of lncRNA CASC9. Therefore, we conclude that CASC9 relieves the inhibition of STAT3 induced by miR‐519d to recover STAT3 expression, reversely, STAT3 could activate the transcription activity of CASC9, forming the positive feedback loop of STAT3‐CASC9‐miR‐519d‐STAT3 axis. STAT3 functions as a vital transcription factor in the pathophysiological process. For the gliomagenesis, STAT3 is found to be an important oncogenic driver via constitutively activated in glioma‐initiating cells by phosphorylation on both tyrosine (Y705) and serine (S727) residues.^30^

Overall, these data and finding could powerfully support the conclusion that CASC9 promotes the glioma tumourigenesis in vitro and in vivo. Mechanically, CASC9 sponges miR‐519d to resurgence STAT3 inhibition, then STAT3 transcription factor in return activate CASC9. The new findings of molecular mechanism provide a valuable therapeutic strategy for the glioma treatment.

## CONFLICT OF INTEREST

All authors declare no conflicts of interest.
